# Risk of Intraoperative Periprosthetic Femoral Fracture According to Cementless Femoral Stem Geometry: A Cohort Study Based on the Radaelli Classification

**DOI:** 10.7759/cureus.106425

**Published:** 2026-04-04

**Authors:** Jorge A Izquierdo, Jacobo Kerbel, Gilberto Apodaca, Iturbide A Ponce de Leon Sandoval, Regina Diaz, Carlos R Luna, Rodrigo Anaya, Javier Camacho-Galindo

**Affiliations:** 1 Orthopaedics and Traumatology, Centro Médico American British Cowdray (ABC), Mexico City, MEX

**Keywords:** cementless femoral stem, femoral stem geometry, periprosthetic femoral fracture, radaelli classification, total hip arthroplasty (tha)

## Abstract

Background

Intraoperative periprosthetic femoral fracture is an uncommon but clinically significant complication of total hip arthroplasty (THA) that may compromise implant stability and increase the risk of early revision surgery. Several patient- and surgery-related factors have been associated with its occurrence, including bone quality, femoral morphology, and implant design. Cementless femoral stems have been associated with a higher incidence of intraoperative fractures compared with cemented fixation due to the need to achieve press-fit stability and increased implant-bone contact during implantation. Recently, a classification system proposed by Radaelli and colleagues categorized cementless femoral stems according to geometry, length, and modularity, allowing a standardized comparison between implant designs. However, limited evidence exists evaluating the association between femoral stem geometry according to this classification and the risk of intraoperative periprosthetic fracture.

Methods

A retrospective cohort study was conducted, including patients who underwent primary THA with an uncemented femoral component between January 2019 and March 2025 at a tertiary care hospital in Mexico City. Patients were identified through the institutional surgical database using the International Statistical Classification of Diseases and Related Health Problems 10th Revision (ICD-10) procedural codes. Demographic, clinical, radiographic, and surgical variables were collected from electronic medical records. Femoral stem geometry was classified according to the Radaelli classification. The primary outcome was the occurrence of intraoperative femoral periprosthetic fracture. Statistical analysis included descriptive statistics, group comparisons, and logistic regression to evaluate associations between stem geometry and fracture risk.

Results

A total of 1,384 patients undergoing primary THA with an uncemented femoral stem were included. Fifty intraoperative femoral periprosthetic fractures were identified, corresponding to an incidence of 3.6%, which falls within the range reported in previous studies. The most frequently used femoral stem geometries were short fit-and-fill stems (455, 32.9%), flat wedge stems (396, 28.7%), and quadrangular wedge stems (188, 13.6%). Flat wedge stems accounted for the highest proportion of fractures (17, 34%), followed by short fit-and-fill stems (10, 20%). Logistic regression analysis demonstrated no statistically significant association between most stem geometries and fracture risk. The only geometry associated with increased risk was C2 fit-and-fill stems (OR 22.29; 95% CI 1.34-371.82); however, this finding was based on a very small sample size.

Conclusion

No statistically significant association was identified between most femoral stem geometries according to the Radaelli classification and fracture risk. Flat wedge stems accounted for the highest proportion of fractures in this series, although without demonstrating an independent statistical association.

## Introduction

Total hip arthroplasty (THA) is one of the most successful surgical procedures in modern medicine and continues to grow rapidly worldwide. Because of its excellent clinical outcomes and improvement in quality of life, THA has been described as the "operation of the century" [1]. The number of primary and revision procedures is projected to increase substantially over the coming decades, with estimates suggesting a rise of approximately 71% in primary procedures and between 43% and 70% in revision procedures by 2030 in the United States [2-4]. As procedure volume continues to rise, understanding the complications associated with THA and the factors that influence their development has become increasingly important.

Intraoperative periprosthetic femoral fracture is an uncommon but clinically significant complication of THA. Although its incidence is relatively low, its occurrence may compromise implant stability, increase operative time, and potentially lead to early implant failure or the need for revision surgery [5,6]. Furthermore, the management of these fractures may increase healthcare costs and place an additional burden on healthcare systems [7].

Multiple patient-related and surgical factors have been associated with the development of intraoperative periprosthetic femoral fractures. Patient-related factors include age, sex, bone quality, and femoral morphology. The risk associated with age has been described as bimodal, with higher incidence in patients younger than 50 years and older than 80 years. Morphological factors of the proximal femur, including neck-shaft angle, preservation of femoral neck length, canal flare index, and bone mineral density, have also been associated with fracture risk. Surgical variables such as implant design, canal preparation techniques, including the use of automated instrumentation, and surgeon-related factors, such as training in arthroplasty surgery and surgical volume, have also been identified as contributors [8-10].

The type of femoral fixation used during THA also plays an important role. Several studies have demonstrated that cementless femoral stems may be associated with a higher incidence of intraoperative periprosthetic femoral fractures compared with cemented stems, largely due to the need to achieve primary stability through press-fit fixation and increased implant-bone contact during implantation [11-14].

Over the past decades, the number of available cementless femoral stem designs has increased significantly, reflecting ongoing efforts to improve implant fixation and long-term survivorship. As a result, several classification systems have been proposed to categorize femoral stems according to their design characteristics and fixation patterns [15,16].

More recently, Radaelli and colleagues introduced a classification system for cementless femoral stems that categorizes implants according to three main characteristics, namely, stem geometry, stem length, and modularity, with additional modifiers including collar presence and surface technology [17]. This classification provides a standardized framework for evaluating contemporary femoral stem designs and facilitates comparisons between different implant geometries. Understanding the relationship between femoral stem geometry and intraoperative fracture risk may have direct clinical implications, as it can support preoperative planning, implant selection, and risk stratification, particularly in patients with unfavorable femoral morphology or compromised bone quality.

This study aimed to evaluate the association between cementless femoral stem geometry, as classified by the Radaelli system, and the risk of intraoperative periprosthetic femoral fracture in primary THA.

## Materials and methods

**Figure 1:**
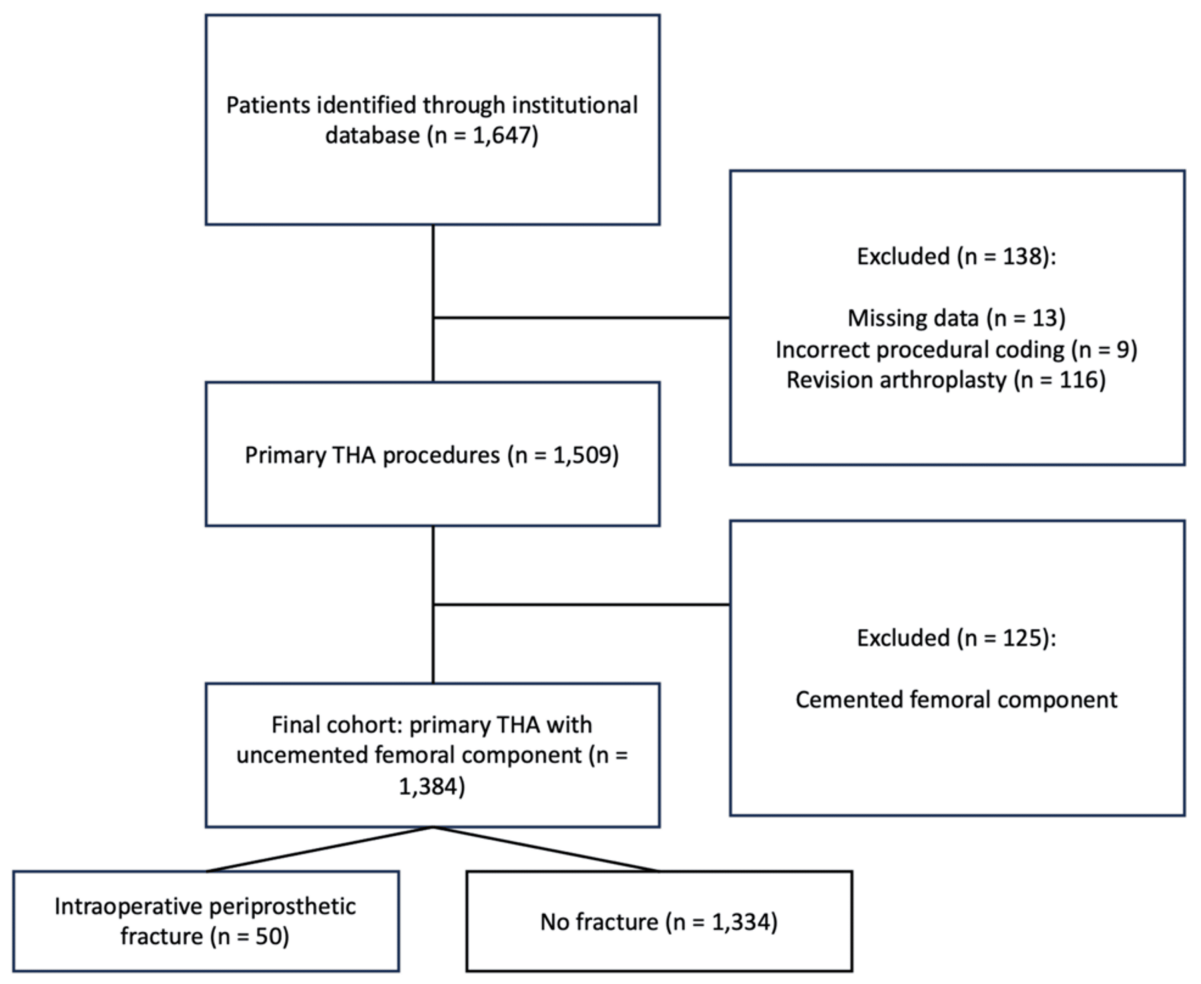
Flowchart of patient selection A total of 1,647 patients with procedural coding for hip arthroplasty were identified. After applying the exclusion criteria, 1,384 patients undergoing primary THA with an uncemented femoral component were included in the final analysis. The incidence of intraoperative periprosthetic femoral fracture was 3.6%. The image was created by the authors using PowerPoint (Microsoft Corporation, Redmond, Washington, United States). THA: total hip arthroplasty

A retrospective cohort study was conducted including patients who underwent primary THA with an uncemented femoral component between January 2019 and March 2025 at both campuses (Observatorio and Santa Fe) of Centro Médico American British Cowdray (ABC), a private tertiary care hospital system in Mexico City. Patients were identified through the institutional surgical database using the International Statistical Classification of Diseases and Related Health Problems 10th Revision (ICD-10) coding system, specifically procedural codes corresponding to hip arthroplasty procedures (05R9, 05RA, 05RB, 05RE, 05RR, and 05R5). A total of 1,647 patients were initially identified; after applying the predefined inclusion and exclusion criteria, the final cohort consisted of 1,384 patients (Figure 1).

Patients were included if they underwent primary hip arthroplasty during the study period and had a complete electronic medical record available in the institutional systems (OnBase, TIMSA electronic medical record, and Vue Motion PACS). Exclusion criteria included cemented femoral stems, revision hip arthroplasty procedures, absence of a postoperative control radiograph obtained on the day of surgery, femoral preparation performed using automated devices, incorrect procedural coding, or incomplete medical records.

Sample size estimation was performed using OpenEpi (Dean AG, Sullivan KM, Soe MM. OpenEpi: Open Source Epidemiologic Statistics for Public Health, Version. www.OpenEpi.com, updated 2013/04/06) with the formula for cohort studies assuming a 95% confidence level, an 80% statistical power, an exposure ratio of 1, an outcome prevalence of 1.6%, and an expected odds ratio of 2.7 according to previous literature [18]. Using the uncorrected Fleiss formula, the estimated minimum sample size was 1,302 patients.

Demographic, clinical, radiographic, and surgical variables were collected from the medical records. These included age, sex, laterality, weight, height, body mass index, preoperative diagnosis, comorbidities, preoperative Harris hip score, Kellgren-Lawrence classification for osteoarthritis, and Ficat classification for avascular necrosis [19-21]. Perioperative variables included hospital campus, operative time, estimated blood loss, length of hospital stay, and surgical approach (posterior, lateral, or anterior). Implant-related variables included femoral stem geometry according to the Radaelli classification [17].

Femoral stem geometry was classified according to the Radaelli classification based on previously published categorizations of implant models and manufacturers, as described in the original and subsequent studies.

The primary outcome was the occurrence of intraoperative periprosthetic femoral fracture. Intraoperative periprosthetic femoral fracture was defined as a disruption of femoral cortical continuity occurring in relation to implant placement during the perioperative period, based on intraoperative documentation and immediate postoperative radiographic findings. When fractures occurred, they were classified using the Vancouver intraoperative classification, the Unified Classification System for Periprosthetic Fractures (UCPF), and the Mallory classification [22,23]. Additional variables included the moment of fracture identification, treatment used for fracture management, and postoperative weight-bearing protocol.

Statistical analysis was performed using IBM SPSS Statistics for Windows, Version 27.0 (IBM Corp., Armonk, New York, United States). Continuous variables were reported as mean and standard deviation or median and interquartile range according to distribution, while categorical variables were expressed as frequencies and percentages. Normality was assessed using the Kolmogorov-Smirnov test. Group comparisons were performed using Student's t-test or Mann-Whitney U test for continuous variables and the chi-squared test for categorical variables. Risk analysis was performed using relative risks for 2×2 contingency tables and odds ratios derived from binary logistic regression. Binary logistic regression analysis was performed to evaluate the association between femoral stem geometry and intraoperative periprosthetic fracture. Femoral stem geometry was included as the primary independent variable, and geometry A was used as the reference category, as it represented the group with the highest number of observed fractures. Statistical significance was defined as p<0.05.

The present study is derived from an institutional cohort that has also been analyzed in a previously published investigation evaluating the association between surgical approach and intraoperative periprosthetic femoral fracture [24]. However, the current analysis addresses a distinct research question focused specifically on femoral stem geometry.

The study protocol was approved by the Research Committee and Research Ethics Committee of Centro Médico American British Cowdray (ABC) (approval number: CMABC-25-35). Because the study consisted of a retrospective review of clinical and radiographic records, it was considered minimal risk for participants, and all data were handled confidentially.

## Results

The final cohort included 1,384 patients who underwent primary THA with an uncemented femoral stem. Among these patients, 50 intraoperative femoral periprosthetic fractures were identified, corresponding to an overall incidence of 3.6%.

The median age of the overall population was 70 years, with 475 (34.3%) male and 909 (65.7%) female patients. The median body weight was 68 kg, median height 165 cm, and median body mass index 24.9 kg/m². In the group that developed intraoperative periprosthetic fracture, the median age was 69 years, with 34 (68%) female and 16 (32%) male patients. The median weight in this group was 66.5 kg, median height 162 cm, and median body mass index 25 kg/m².

When comparing patients with and without fractures, the only baseline variable that demonstrated a statistically significant difference was patient height (p=0.01). Surgical time was also significantly longer in the fracture group (p=0.01). No statistically significant differences were observed for age, sex, weight, body mass index, length of hospital stay, or estimated blood loss (Table 1).

**Table 1:** Baseline characteristics of patients with and without intraoperative periprosthetic femoral fracture Values are presented as median (IQR) or n (%). Group comparisons were performed using the Mann-Whitney U test for continuous variables and the chi-squared test for categorical variables, and the p-value is considered significant if p<0.05. ^a^ indicates that the chi-squared test was used. IQR: interquartile range; BMI: body mass index

Variable	No fracture (n=1,334)	Fracture (n=50)	χ²	P-value
Age, median (IQR), years	70 (61-78)	69 (60-78)	-	0.51
Sex				
Male, n (%)	459 (33.2)	16 (32)	0.124^a^	0.73
Female, n (%)	875 (65.6)	34 (68)		
Weight, median (IQR), kg	68 (60-80)	66.5 (58-78.5)	-	0.35
Height, median (IQR), cm	165 (160-171)	162 (154-169)	-	0.01
BMI, median (IQR), kg/m²	24.9 (22.8-27.6)	25 (22.8-27.6)	-	0.66
Length of hospital stay, median (IQR), days	4 (3-5)	4 (3-5)	-	0.47
Surgical time, median (IQR), minutes	135 (120-180)	157 (120-210)	-	0.01
Estimated blood loss, median (IQR), mL	400 (250-500)	400 (300-637)	-	0.10

The most common preoperative diagnosis was coxarthrosis or chondral pathology in 932 patients (67.3%), followed by hip fracture in 393 patients (28.4%) and avascular necrosis of the femoral head in 49 patients (3.5%) (Table 2).

**Table 2:** Preoperative diagnosis of the study population Values are presented as n (%). Group comparisons were performed using the chi-squared test for categorical variables, and the p-value is considered significant if p<0.05.

Diagnosis	Total (N=1,384)	No fracture (n=1,334)	Fracture (n=50)	P-value	χ²
Coxarthrosis/chondral pathology	932 (67.3%)	901 (67.5%)	31 (62%)	<0.05	29.944
Hip fracture	393 (28.4%)	381 (28.6%)	12 (24%)	>0.05	0.493
Avascular necrosis	49 (3.5%)	45 (3.4%)	4 (8%)	>0.05	3.021
Dysplasia sequelae	8 (0.6%)	5 (0.4%)	3 (6%)	<0.05	26.536
Tumor	2 (0.1%)	2 (0.1%)	0 (0%)	>0.05	0.075

Comparative analysis by diagnosis demonstrated that sequelae of developmental dysplasia of the hip were associated with a significantly higher risk of intraoperative periprosthetic fracture compared with coxarthrosis. In contrast, hip fracture as a preoperative diagnosis did not demonstrate a statistically significant increase in intraoperative fracture risk in this cohort.

Regarding implant characteristics, the most frequently used femoral stem geometries according to the Radaelli classification were short fit-and-fill stems (C3) in 455 patients (32.9%), flat wedge stems (A) in 396 patients (28.7%), and quadrangular wedge stems (B2) in 188 patients (13.6%).

The geometry associated with the highest absolute number of intraoperative fractures was flat wedge stems (geometry A), accounting for 17 cases (34%), followed by short fit-and-fill stems (C3) with 10 cases (20%) (Table 3).

**Table 3:** Distribution of femoral stem geometry according to the Radaelli classification Values are presented as n (%). Stem geometry was classified according to the Radaelli classification. Group comparisons were performed using the chi-squared test for categorical variables, and the p-value is considered significant if p<0.05. ^a^ indicates that the chi-squared test was used.

Stem geometry	Total (N=1,384)	No fracture (n=1,334)	Fracture (n=50)	P-value	χ²
A	396 (28.7%)	379 (28.4%)	17 (34%)	<0.01 (after excluding C2 geometry, p=0.306)	20.784^a^ (8.307^a^)
B1	8 (0.6%)	8 (0.6%)	0 (0%)		
B2	188 (13.6%)	183 (13.7%)	7 (14%)		
B3	27 (2%)	24 (1.8%)	3 (6%)		
C1	184 (13.3%)	177 (13.3%)	7 (14%)		
C2	2 (0.1%)	1 (0.1%)	1 (2%)		
C3	455 (32.9%)	445 (33.4%)	10 (20%)		
D	79 (5.7%)	76 (5.7%)	3 (6%)		
E	44 (3.2%)	42 (3.1%)	2 (4%)		

Binary logistic regression analysis demonstrated no statistically significant association between most femoral stem geometries and the risk of intraoperative periprosthetic fracture. The only geometry associated with increased risk was C2 fit-and-fill stems (OR 22.29; 95% CI 1.34-371.82; p=0.03). However, this finding was based on a very small sample size, as only two implants were classified as C2, one of which developed a fracture (Table 4).

**Table 4:** Logistic regression analysis of femoral stem geometry and risk of intraoperative periprosthetic fracture Odds ratios were calculated using binary logistic regression, with geometry A (flat wedge stems) used as the reference category. The p-value is considered significant if p<0.05.

Stem geometry (vs. geometry A)	OR	95% CI	P-value
B1	-	-	1.00
B2	0.73	0.29-1.90	0.52
B3	2.79	0.76-10.17	0.12
C1	0.88	0.36-2.16	0.78
C2	22.29	1.34-371.82	0.03
C3	0.50	0.23-1.11	0.09
D	0.88	0.25-3.08	0.84
E	1.11	0.25-5.00	0.89

After excluding this subgroup from the analysis due to its limited sample size, no statistically significant association was identified between any femoral stem geometry and fracture risk.

Among the fractures identified, the most frequent pattern according to the Vancouver intraoperative classification was type A2, observed in 39 cases (78%). According to the UCPF, the most frequent type was B1 in 35 cases (70%), while the Mallory classification most commonly identified type I fractures in 21 cases (52.5%).

The most common intraoperative moment of fracture identification was during femoral canal preparation in 22 cases (44%), followed by final stem implantation in 20 cases (40%). In three patients, the fracture was identified on immediate postoperative radiographs.

## Discussion

In this study, 50 intraoperative femoral periprosthetic fractures were identified among 1,384 patients undergoing primary THA with an uncemented femoral stem, representing an incidence of 3.6%. This finding falls within the wide range reported in the literature, although it is slightly higher than the incidence described in large registry-based studies [6,25].

Previously described risk factors for intraoperative periprosthetic fractures include patient-related and surgical variables. Patient-related factors such as age, sex, bone quality, and femoral morphology have been consistently associated with fracture risk [8-10]. The association with age has been described as bimodal, with increased risk observed in patients younger than 50 years and older than 80 years [9]. Morphological characteristics of the proximal femur, including neck-shaft angle, preservation of femoral neck length, canal flare index, and bone mineral density, have also been associated with fracture susceptibility [8-10].

In our study, most baseline demographic variables did not demonstrate a statistically significant association with fracture risk. The only variable that showed a significant difference was patient height, which was slightly lower in patients who developed fractures. Although the clinical relevance of this finding is uncertain, it may reflect differences in femoral morphology or implant sizing.

Preoperative diagnosis demonstrated a significant association with fracture risk. Patients with sequelae of developmental dysplasia of the hip had a markedly increased risk of intraoperative periprosthetic fracture compared with those undergoing surgery for coxarthrosis. This finding likely reflects altered proximal femoral anatomy, including variations in canal geometry and bone stock, which may complicate implant positioning and increase susceptibility to fracture. In contrast, hip fracture as a preoperative diagnosis did not demonstrate an increased risk of intraoperative fracture in this cohort.

The primary objective of this study was to evaluate the association between femoral stem geometry according to the Radaelli classification and the risk of intraoperative periprosthetic fracture [17]. Although flat wedge stems accounted for the highest absolute number of fractures, no statistically significant association was identified between most stem geometries and fracture risk after adjustment. The only statistically significant finding corresponded to C2 fit-and-fill stems; however, this result was based on a very small sample size and should be interpreted with caution.

These findings suggest that, within the context of contemporary implant designs, femoral stem geometry alone may not be an independent determinant of intraoperative fracture risk. However, given the relatively low event rate and the limited sample size within individual geometric subgroups, a larger study would be required to adequately determine whether stem geometry represents an independent risk factor. Fracture occurrence is likely multifactorial and influenced by the interaction between implant characteristics and patient-specific anatomical factors [26-30].

From a surgical perspective, intraoperative fractures were most frequently identified during femoral canal preparation and final implant insertion. This observation supports the concept that fracture risk is closely related to the mechanical stresses generated during press-fit implantation, particularly when adequate proximal support is not achieved [22,23].

The distribution of fracture patterns in our study was consistent with previous reports. The majority of fractures were classified as stable metaphyseal injuries, most commonly Vancouver type A2, which are typically managed with cerclage fixation [22,23].

This study has several limitations. First, its retrospective design introduces potential sources of bias and limits the ability to establish causal relationships. Second, the relatively low incidence of intraoperative fractures reduces statistical power, particularly when analyzing multiple stem subgroups, resulting in wide confidence intervals for some estimates. Third, important variables such as proximal femoral morphology, including Dorr classification or cortical thickness indices, were not evaluated. This was primarily due to the lack of standardized preoperative radiographic studies, as many patients underwent imaging outside the institution, limiting the ability to perform consistent radiographic measurements.

Additionally, although variables such as surgical approach and surgeon-related factors may influence fracture risk, these were not the focus of the present analysis. These factors are currently being evaluated in separate studies derived from the same institutional cohort.

Despite these limitations, this study includes a large cohort from a single institution and reflects real-world clinical practice, including a wide variety of implant designs. The use of a contemporary classification system such as the Radaelli classification provides a standardized framework for evaluating femoral stem geometry and its potential association with intraoperative complications.

## Conclusions

In this study, the incidence of intraoperative femoral periprosthetic fracture during primary THA with uncemented femoral stems was within the range reported in the literature. No statistically significant association was identified between most femoral stem geometries according to the Radaelli classification and fracture risk. Although flat wedge stems accounted for the highest proportion of fractures in this cohort, no independent association was demonstrated.

Patient-related factors affecting proximal femoral morphology, particularly developmental dysplasia of the hip, as well as differences in patient height, may influence the risk of intraoperative fracture and should be considered during preoperative planning and implant selection. Most fractures occurred during femoral canal preparation or final stem implantation and were predominantly stable metaphyseal fractures, consistent with previously described patterns.

Given the limited number of events within specific geometric subgroups, larger studies are required to determine whether femoral stem geometry represents an independent risk factor for intraoperative periprosthetic fracture. These findings support a multifactorial understanding of fracture risk and highlight the need for further investigation using adequately powered cohorts.
